# Similarity Analysis of Computer-Generated and Commercial Libraries for Targeted Biocompatible Coded Amino Acid Replacement

**DOI:** 10.3390/ijms252212343

**Published:** 2024-11-17

**Authors:** Markus Meringer, Gerardo M. Casanola-Martin, Bakhtiyor Rasulev, H. James Cleaves

**Affiliations:** 1 German Aerospace Center (DLR), Department of Atmospheric Processors, Oberpfaffenhofen, 82234 Wessling, Germany; markus.meringer@dlr.de; 2 Department of Coatings and Polymeric Materials, North Dakota State University, Fargo, ND 58108, USA; gerardo.casanolamart@ndsu.edu (G.M.C.-M.); bakhtiyor.rasulev@ndsu.edu (B.R.); 3 Department of Chemistry, National University of Uzbekistan, Tashkent 100174, Uzbekistan; 4 Department of Chemistry, Howard University, Washington, DC 20059, USA; 5 Earth-Life Science Institute, Tokyo Institute of Technology, 2-12-1-IE-1 Ookayama, Meguro-ku, Tokyo 152-8550, Japan; 6 Blue Marble Space Institute for Science, 1001 4th Ave, Suite 3201, Seattle, WA 98154, USA

**Keywords:** amino acid libraries, structural similarity, molecular fingerprint, Tanimoto coefficient, multi-dimensional scaling

## Abstract

Many non-natural amino acids can be incorporated by biological systems into coded functional peptides and proteins. For such incorporations to be effective, they must not only be compatible with the desired function but also evade various biochemical error-checking mechanisms. The underlying molecular mechanisms are complex, and this problem has been approached previously largely by expert perception of isomer compatibility, followed by empirical study. However, the number of amino acids that might be incorporable by the biological coding machinery may be too large to survey efficiently using such an intuitive approach. We introduce here a workflow for searching real and computed non-natural amino acid libraries for biosimilar amino acids which may be incorporable into coded proteins with minimal unintended disturbance of function. This workflow was also applied to molecules which have been previously benchmarked for their compatibility with the biological translation apparatus, as well as commercial catalogs. We report the results of scoring their contents based on fingerprint similarity via Tanimoto coefficients. These similarity scoring methods reveal candidate amino acids which could be substitutable into modern proteins. Our analysis discovers some already-implemented substitutions, but also suggests many novel ones.

## 1. Introduction

Modern biology uses 20 canonical coded amino acids (CAAs); however, there are occasional coded substitutions (e.g., selenocysteine and pyrrololysine [1], and many other non-canonical amino acids have been engineered or otherwise substituted into proteins [2]. It is believed that early organisms used simpler and more promiscuously coded amino acid sets, and that the tolerance of biological proteins and the processes that makes them into dissimilar coded amino acids has changed over time [3,4,5]. Since tRNA amino acid charging is accomplished and error-checked by proteins, this reduction in ambiguity occurred in tandem with the development of the biological machinery which accomplishes it. This machinery developed in the context of the compounds present in evolving organisms, and there are a great number of possible isomers of any given amino acid, many of which are real metabolites. Indeed, organisms have evolved various secondary metabolite amino acid analogues which can interfere with protein biosynthesis in predators or competitors and are not recognized by their own translation machinery [6].

Proteins, and other biological macromolecules, can tolerate multiple xenobiological monomer substitutions (see, for example, Marlière et al. [7], Malyshev et al. [8]). This is mainly due to the way in which biological information is communicated in molecules, e.g., a combination of shape and surface electron density interactions between macromolecules and small molecules governs the strength of molecular interaction [9]. For this reason, drugs are effective modulators of enzymatic activity, and molecular similarity is a powerful tool for finding them [10].

Biological information encoding, which entails the reliable connection of structural interaction and kinetic information with downstream biological “meaning,” is mediated by several processes involved in translation, including the interactions of amino acids and aminoacyl-tRNA synthetases during tRNA charging and the interactions of aminoacyl-tRNAs with the ribosome during translation. Furthermore, as it is likely that the genetic code has evolved over time [3,5], this interfacial “language” has had to adapt to the ways that the incorporation of new structural elements enabled new coding dynamics over time.

Organic chemical structure space is vast [11]. This has implications for why biology uses the compounds it does [12]. Biology’s choice of components is directed and constrained by its composition at any given point in time [13,14]. This is analogous to the development of languages and syntaxes: initially poorly constrained relational systems which interact by feedback with mechanisms which enforce their existence can rapidly reinforce canonical encodings [15]. However, such analyses are likely blind to interfering noise which does not exist in the learning system during its development. Such noise can be introduced later into such systems and potentially tolerated well. This dynamic likely lies at the heart of biological evolution.

As an example of a non-engineered incorporation of a non-canonical amino acid into biological proteins, it has been shown that norleucine (norleu) can substitute for methionine (M) in proteins due to their molecular similarity [2]. The CH_2_CH_2_CH_3_ structural motif in norleu is apparently similar enough to M’s CH_2_SCH_3_ motif that this compound evades protein synthesis error-correction mechanisms and becomes incorporated in biological proteins using the same coding mechanisms. However, norleu is not a major intermediate in any known biosynthetic pathway, and thus, most cells never experience situations in which they must carefully distinguish between M and norleu. In this case, norleucine may be functionally substitutable, tolerably substitutable with reduced function, or a complete poison for any given protein, but this situation rarely arises.

Compounds which are structurally compatible because their functional groups do not interfere with normal biological interactions are known as bioisosteres [16]. Bioisosteres may be rather common in chemical space; this study aims to provide a novel method to find them.

Amino acids are especially attractive targets for bioisostere exploration because of their fundamental roles in biochemistry. In addition to amino acid analogues which may serve as enzyme agonists or antagonists, coded amino acid analogues can modulate coded protein function [17], and amino acids are important intermediates in many biosynthetic pathways as well as important neurotransmitters (e.g., GABA, glutamic acid (E), aspartic acid (D), serine (S), glycine (G) [18]). There have been a few reported screens examining the promiscuity of the translation apparatus, which examined scores of purchasable amino acids, including ɑ-, β-, and ɑ,ɑ-substituted amino acids [19,20].

Introducing new monomers in biopolymers often depends on finding compounds with similar shape and electrotopological properties. This could be especially informative in the case of coded amino acids used in biopolymers which may have multiple levels at which their compatibility is proofread by biological processes. For amino acid incorporation in proteins, amino acids must be recognized by tRNA synthetases, then be tolerated by the ribosomal machinery, and finally not interfere too strongly with the resulting protein’s folding and function. While incorporation of a single residue stochastically at any given site in a protein sequence may be tolerable, multiple or complete substitution may not be. Some sites may also be more sensitive to substitutions than others, or particular types of substitutions may be problematic for many reasons. In the case of amino acids present in the growth medium, there must also be mechanisms for importation of the molecules inside the cell. Thus, substitution can fail at many levels.

There are multiple computational methods already developed for performing such searche [21,22,23]. Our approach is novel in its use of the combination of structure enumeration and similarity metrics, and its application to amino acid structures. The similarity and biocompatibility of amino acids are important to analyze to understand possible prebiotic interactions between peptides and other molecules. In a previous study [24], we generated virtual libraries of amino acids using MOLGEN 5.0 structure generation software [25,26]. These sets were designed starting from molecular formulas and sub-formulas of the 20 CAAs using lists of implausible substructures to exclude sterically and energetically unstable molecules. This resulted in a virtual library surrounding the chemical space defined by the 20 CAAs. This dataset was primarily developed for exploration of the evolution of the genetically encoded amino acids [13,14,27,28]) and we use it here to search for potentially biocompatible amino acids.

## 2. Results and Discussion

### 2.1. Composition of the Library

**Figure 1:**
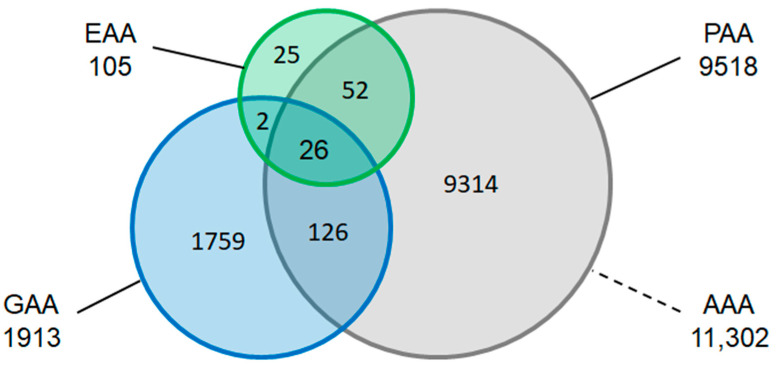
Venn diagram of EAAs, GAAs, and PAAs.

After removal of duplicate structures, applying the procedure described in the Section 3, we arrived at a total of 11,302 structures. Table 1 contains the sizes of each set, and Figure 1 shows a Venn diagram of the overlap of EAAs, GAAs, and PAAs, which underscores the notion that this space has not been extensively explored to date. The SI contains a comma-separated value (.csv) file with SMILES representations of all 11,302 AAs in the rows and columns indicating the set memberships of each structure.

### 2.2. Tanimoto Coefficients

**Figure 2:**
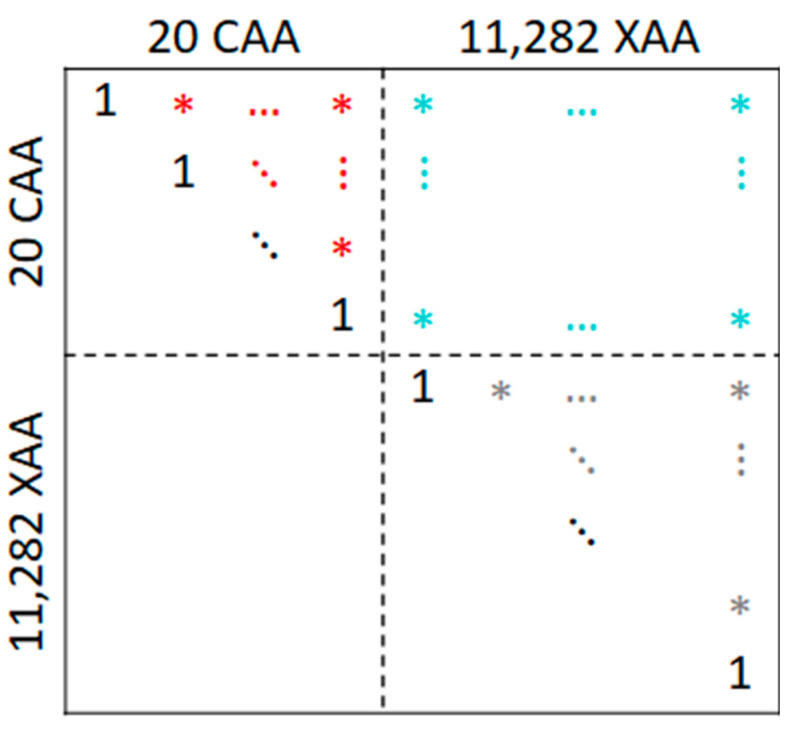
Block structure of the similarity matrix. Asterisks represent arbitrary, non-diagonal matrix entries.

**Figure 3:**
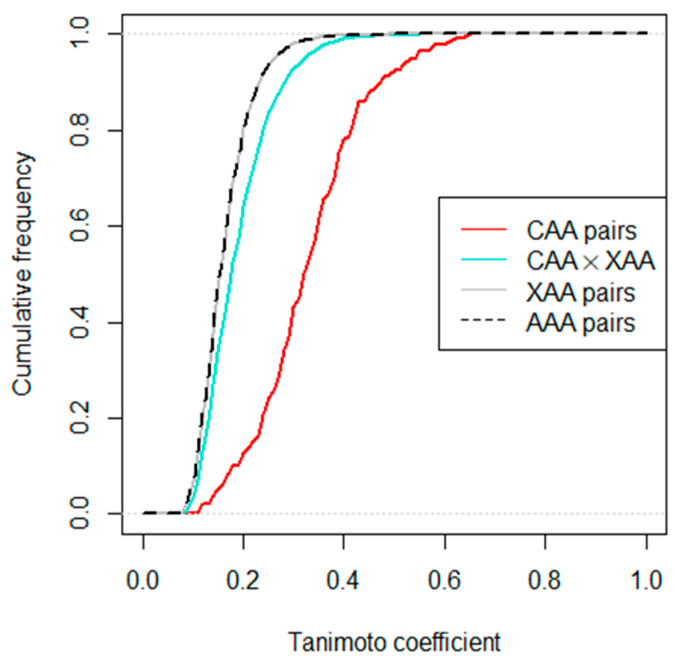
Cumulative relative frequencies of Tanimoto coefficients for different parts of the similarity matrix. For more explanation, see Section 2.2.

TCs are stored in a square matrix of 11,032 rows and columns, which we call the similarity matrix. This matrix is symmetric because, for two AAs X and Y, we have TC(X, Y) = TC(Y, X), and there are entries of 1 on the main diagonal, because TC(X, X) = 1. In Figure 2, which shows the block structure of the similarity matrix, this is depicted by entries of 1 on the main diagonal, and arbitrary entries in the upper triangle matrix represented by asterisks. We will focus on this triangle matrix to avoid counting TCs of the same pair of AAs twice. This triangle matrix has 11,302 ∙ 11,301/2 = 63,861,951 entries. The black stroke curve of Figure 3 shows cumulative relative frequencies of these entries; Figure S1 (bottom left) of the SI shows the distribution as a histogram, and the last column of Table 2 reports key statistical parameters for these TCs.

Focusing on the CAA block of the similarity matrix, the upper triangle matrix has 20 ∙ 19/2 = 190 entries. These entries are highlighted in red in Figure 2; cumulative relative frequencies are depicted in the red curve in Figure 3. A histogram of these TCs among pairs of CAAs is shown in the top left panel of Figure S1, and key statistical parameters are reported in column CAA of Table 2.

Of particular interest for this study is the top right block of the similarity matrix, with entries colored in turquoise in Figure 2. This block contains all TCs of pairs composed of a CAA and an XAA. It has 20 ∙ 11,282 = 225,640 entries; cumulative relative frequencies are depicted by the turquoise curve in Figure 3, and histograms are shown in the right panels of Figure S1. We refer to these TCs as CAA × XAA, denoting that here, the similarity for all pairs of the Cartesian product of CAA and XAA are covered.

Comparing the cumulative relative frequencies of Figure 3, we notice that TCs of AAAs are tendentially slightly lower than those of CAA × XAA, while those of CAAs are in turn somewhat higher than those of CAA × XAA. This can, as well, be seen by comparing the histograms of Figure S1, and is also numerically confirmed in Table 2. The first and the third quartiles, as well as the reported quantiles, median, and mean are lowest for AAA and highest for CAA TCs. It is also interesting to note that the AAA TCs have a maximum value of 1, meaning there must be pairs of structures with identical fingerprints among our AAs (cf. SI section *Choice of Fingerprints* and Figure S2).

Table 3 shows a detailed view of all TCs for CAAs. Columns and rows are sorted with respect to increasing molecular weight and labeled with one-letter CAA abbreviations. Table cells are color-coded by a red–yellow–green scale, with red corresponding to low, and green to high, TC. The red colors in the row and column P indicate the low similarity of proline with all other CAAs, which can be explained by P’s unique cyclic amino acid backbone. Looking for further regions of low similarity, we see that the low-weight AAs G, alanine (A), valine (V), and threonine (T) have low TC, with the high-weight AAs histidine (H), phenylalanine (F), arginine (R), tyrosine (Y), and tryptophan (W), resulting in orange-colored, rectangular-shaped areas in the upper right and lower left corners of the CAA similarity matrix. This pattern is only interrupted by some yellow-colored cells in the row and column of serine (S). The maximum TC of 0.65 among CAAs is met by V and Y. We will use this value of 0.65 later as a threshold for presenting the most similar XAAs for each CAA. Further highly similar pairs of CAAs are A and V, with TC(A, V) = 0.611; asparagine (N) and aspartic acid (D), with TC(N, D) = 0.636; and glutamine (Q) and glutamic acid (E), with TC(Q, E) = 0.607. Figure S3 shows cumulative relative frequencies of TCs for each of the 20 CAAs as color-coded curves, and in SI section *TC Outliers and Choice of TC Threshold*, we show an outlier analysis of TCs of CAA × XAA with respect to the 20 CAAs using box plots (Figure S4). We find that there are a total of 566 extreme outliers at the high TC end, which would be promising candidates for experimental evaluation. Below, we identify structures of the highest similarity with each CAA.

### 2.3. Ranking

**Figure 4:**
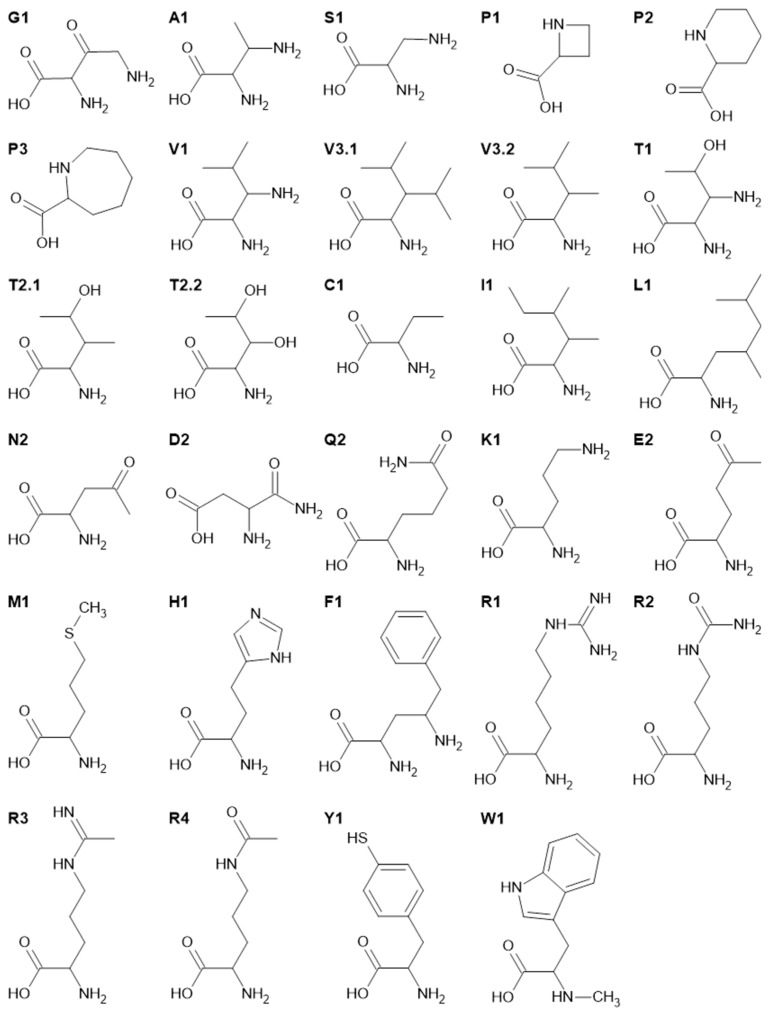
Structures of the most similar XAAs for each CAA.

Using the Tanimoto coefficients, we can sort the structures corresponding to decreasing similarity for each CAA. Figure 4 shows a selection of highly ranked, i.e., very similar, XAAs for each CAA. The following selection rules were applied:

- Report for each CAA the most similar XAA.
- Show further highly ranked XAAs for each CAA with TC greater than 0.65.

For more reasoning behind these rules, the reader is referred to the SI section *TC Outliers and Choice of TC Threshold*.

The structures of Figure 4 have labels composed of a one-letter CAA abbreviation and one or two numbers, e.g., G1 is the highest ranked XAA for glycine and correspondingly A1 for alanine. Table 4 lists, for each CAA, the highest TCs, the corresponding XAA (or CAA) with its abbreviation (ID) from Figure 4, and its set memberships, e.g., G1 has a rather low TC of 0.45 with G. It is included in GAAs, but not in EAAs nor PAAs. For alanine, the highest TC of 0.611 is achieved twice, by A1 and V (omitted in Figure 3, as the CAA structures are generally known). For serine, we again have just a unique highest ranked XAA, S1, with a TC of 0.6. Proline has several analogs with rather high TCs (see Figure S4). The three highest ranked XAAs are labeled P1, P2, and P3 in Figure 4. For V, we have four AAs with TC values above the threshold of 0.65. The highest-ranked structure is labeled, as usual, with V1. The second highest-ranked is identical with A1, i.e., label V2 does not appear in Figure 4 or Table 4. The next highest-ranked structures both have the same TC of 0.667 and thus have labels V3.1 and V3.2 to indicate that both achieve rank three. For T, we have, three times, TC above 0.65, achieved by T1 and the equally scoring T2.1 and T2.2. For cysteine (C), the highest TC is 0.619, i.e., below the threshold of 0.65. Thus, only the highest ranked XAA for C, C1, is depicted in Figure 4. The highest-ranked structure for isoleucine (I) is I1, with a TC of 0.667, and there are no further AAs with TC above 0.65. The most similar AA for leucine (L) is L1, with a TC of 0.643. The highest-ranked structure for N is D and vice versa. We therefore depict the second highest-ranked D2 and N2 in Figure 4. The situation is similar with Q and E, leading to Q2 and E2 shown in Figure 4. The highest-ranked structure for lysine (K) is K1, with a rather high TC of 0.714, and there are no further similar AAs for K with TC above 0.65. The highest-ranked structures for M, H, and F are all below the threshold, and are labeled M1, H1, and F1. For R, the four highest TCs are above 0.65, and the corresponding structures are shown as R1, R2, R3, and R4 in Figure 4. Finally, for Y and W, the highest-ranked AAs again have TCs below 0.65, and the corresponding structures have labels Y1 and W1 in Figure 4.

We note that ornithine (K1) and 1, 3-diaminopropionic acid (S1) are, meanwhile, known to be poor bioisosteres (see e.g., Frenkel-Pinter et al. [29], and Makarov et al. [30]). However, it is not the subject of the present study to review negative experimental results on potential bioisosteres, and we refer to [31] for a comprehensive review of non-canonical tRNA synthetase substrates.

We see in Table 4 that the vast majority of this small selection is commercially available and has not yet been studied experimentally. The SI contains TCs for each CAA, with the entire library as a .CSV file. Further suggestions on how to evaluate AAs with respect to decreasing similarity to a given CAA, and to visualize the most similar analogs, are also offered in the SI. This gives access to a potentially rich set of easily accessible AAs, which may be promising candidates for experimental evaluation.

### 2.4. Projection

**Figure 5:**
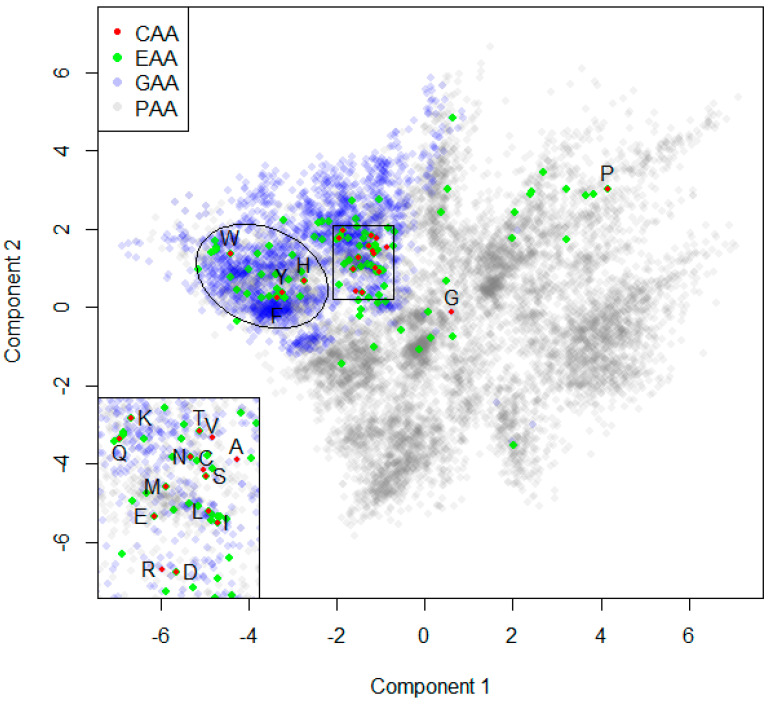
Two-dimensional projection of the AA library using Tanimoto distances and classical MDS.

Figure 5 shows a 2D projection of our AA chemical space using classical MDS with Tanimoto distances as input. We see the smallest CAA, G, taking a central position in this mapping. P is located in the top right region, quite distant from all other CAAs. This again reflects the special structure of P, which we have already noted before when inspecting the matrix of TCs for the CAAs in Table 3. On the left, we see the group of the four aromatic AAs marked by an ellipse. F and Y are located very close to each other, with H also nearby, and W, the largest CAA, a bit further away, at the top left corner of this group. The remaining 14 CAAs are located close to each other in an area marked by a rectangle in Figure 5. The inset at the bottom left corner zooms into this densely populated area. Here, we see closely located pairs of CAAs, such as V and Y (their high TC was mentioned above), C and S (which differ only in their OH/SH group), or the pair of isomers L and I. However, we note that such a projection cannot reflect all similarity relationships perfectly. For instance, N is the most similar CAA for D, but appears at a relatively large Euclidean distance to D in this figure, whereas R, with its rather low TC(R, D) = 0.316, has the shortest Euclidean distance to D among all CAAs in this MDS. There are multiple reasons for these imperfections, particularly the fact that this MDS is calculated to optimize Euclidean distances for all pairs of AAAs, and CAAs are only a tiny subset of AAAs. We present a deeper analysis of the MDS quality in the corresponding SI section. Despite these imperfections, this 2D projection offers a useful alternative to the 1D approach realized by ranking with respect to single CAAs. For instance, using this method, XAAs can be discovered that are not among the top-ranked ones by using the 1D approach, but which are nevertheless interesting due to relatively high simultaneous similarities with multiple CAAs. Another advantage of the 2D projection could be the discovery based on diversity, and to inspect regions not yet covered by EAAs. To simplify access to the structures of XAAs in this projection, we included their 2D coordinates in the .csv file (see SI file AASS.csv). Further recommendations on software to open this .csv file, reproduce the 2D layout, zoom into interesting regions, and display selected structures of interest are also given at the end of the SI section on *Detailed Results*. The quality of our 2D layout is further discussed in the SI section *MDS Quality* and Tables S1–S3 included there.

## 3. Materials and Methods

The set of computationally Generated Amino Acid (GAA) structures used in the present study contains 1913 real and hypothetical compounds, including the 20 CAAs of the standard genetic code, as described in [13]. Additionally, we imported the amino acids tested by Hartman et al. [20] and Josephson et al. [19] via their annotated Chemical Abstracts Service (CAS) number entries as listed on SciFinder Scholar. We refer to this set as Experimentally evaluated Amino Acids (EAAs). We also downloaded amino analogue structures with the HNCCOOH substructure motif under 200 amu from Emolecules (https://www.emolecules.com/ accessed on 2 April 2024) as an .sdf file, and we call this set Purchasable Amino Acids (PAAs).

**Figure 6:**
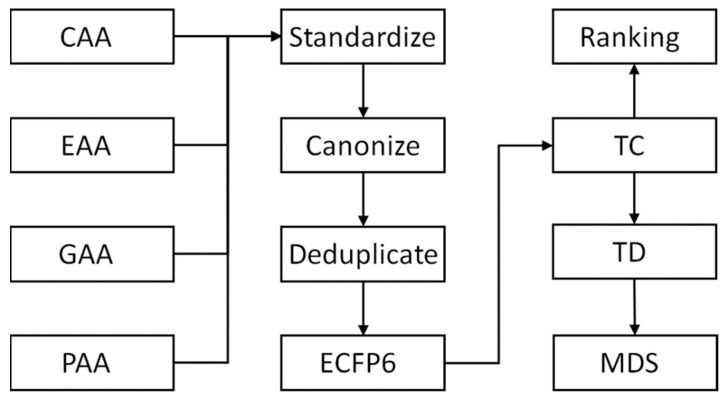
Workflow of the similarity study. For explanation of the abbreviations, see Section 3 and Table 2.

The further processing is sketched in Figure 6. In a standardization step, the PAA set was cleaned of salts, and stereochemical information was removed using the KNIME [32] Chemical Identifier Resolver (CIR) node (AlvaScience, Lecco, Italy). Then, all sets were converted to canonical SMILES using OpenBabel [33]. These were copied into one file and sorted lexicographically, and duplicates were removed. Set memberships of each compound were stored in a table with five columns—SMILES—and columns representing the four sets CAA, EAA, GAA, and PAA. Boolean entries true or false in these columns indicate whether a compound belongs to the set. For this step and all forthcoming computations, we used R version 4.3.1 [34].

Compounds were scored for structural similarity using 2D molecular fingerprints and Tanimoto Coefficients (TCs). For compound representation, we computed Extended Connectivity FingerPrints of diameter 6 (ECFP6s), [35]. ECFP6s are circular fingerprints that encode atom-type information and bond connectivity up to a depth of six non-hydrogen atoms, intending to capture precise atom environment substructural features. The reason for selecting diameter 6 is exposited in the Supplementary Information (SI) section *Choice of Fingerprints*. Fingerprints were computed using the *rcdk* package (version 3.8.1, [36]) starting from SMILES-level representations and resulting in 1024-bit bit-vectors.

TCs [37] were computed among the fingerprint representations with the same R package. They were used as similarity scores and stored in a symmetrical matrix called the similarity matrix. For more mathematical background on TCs, the reader is referred to the SI section *Tanimoto Coefficients*. We sorted the data such that the first rows and columns correspond to CAAs and the latter to non-canonical AAs, which we call Xeno Amino Acids (XAAs). This imposes a block structure to the similarity matrix, which is discussed in detail in Section 2.2. Different blocks of the similarity matrix representing TCs among CAAs, TCs of CAAs with XAAs, and TCs among all amino acids (AAAs) were analyzed using standard statistical parameters (minima, maxima, means, quantiles, etc.). Structures were sorted by decreasing similarity with each of the 20 CAAs. Distributions of TCs were determined using the R function *ecdf* (see Table S2 for the most important R functions used in this study, together with their R package names and version numbers). We conducted an outlier analysis of XAAs based on TCs with CAAs, and the highest-ranked XAAs were determined.

To visualize similarities among AAs, we used classical Multi-Dimensional Scaling (MDS, [38,39,40,41]). MDS algorithms take a distance matrix as input and calculate *n*-dimensional coordinates for each object, i.e., AAs, such that Euclidean distances in *n*-dimensional space optimally represent the input distances. We chose *n* = 2 to represent AAs as a 2-dimensional scatterplot. We used the R function *cmdscale*, which provides the method of Gower [40]. This method is also known as principal coordinate analysis [42]. To convert our similarity matrix given by TCs into a distance matrix, we used the general relationship between similarity and distance [43], i.e., similarity = 1/(1 + distance), and calculated the Tanimoto Distances (TD) as TD = (1—TC)/TC.

We note that TC is always greater than zero in our similarity matrix, so we do not have to face the problem of denominators being zero in this equation.

## 4. Conclusions

These results suggest that there remain numerous untested XAAs that could be substituted into contemporary proteins using the modern translation apparatus. Our original study [24] was limited to amino acids that were most similar to modern biological amino acids, for example, those containing only the elements CHNOS. Many alterations of structure not originally considered in the constructed set are possible, and more nuanced sets could be produced for this sort of comparison [44]. This can be done simply by adapting our previously developed workflow [24]); thus, the current study should be considered a preliminary proof of concept. The inclusion of purchasable AAs in this study sheds some light as to the compositional search space that could be explored (for example, by inclusion of other chemical elements or other formula ranges).

Biological component substitutability likely correlates with both depth of evolutionary biological embeddedness and the permittivity of structure space. For small amino acids, fewer substitutions are possible, simply because the search space is small.

This methodology can likely usefully screen bioactive compounds. Typically, in drug screening, many millions of compounds must be searched to find an active molecule [45]. The CAAs are unlike drugs “in general” in significant ways, and we have screened a much smaller set of compounds than is usually explored in modern drug screening (see for example, The Atomwise AIMS Program [46]).

Amino acids are largely synthesized by organisms themselves, though many higher-trophic-level organisms, including humans, have incomplete amino acid biosynthesis capabilities. This kind of nested interdependency is a common phenomenon among cofactors and primary metabolites [47]. The use of the CAAs likely reflects this dependency. The CAAs also share a common co-evolutionary history (possibly extending back billions of years [3,5]) that has depended on and constrained the types of molecular interactions that were allowed among them as proteins evolved. The CAAs appear to have been evolutionarily selected to be both biosynthetically and functionally “minimal” [5], meaning that they are not only among the lowest-molecular-weight compounds (and therefore have relatively few isomers, as the number of possible isomers grows exponentially with the number of atoms in a molecule [24]) that contain any given functionality, but they are also among the biosynthetically simplest and energetically least costly. Given these deep constraints, it might be surprising if there are many CAA analogues that can be found that can be easily substituted into modern proteins without violating these concepts.

The methodology presented here may also offer a way to screen for mechanisms by which biology arrived at its monomer usage, as compatible bioisosteres or otherwise electrotopologically similar compounds may not be synthetically easily reachable via feasible metabolisms. Thus, there may be an underlying structure to chemical space which guides biology to select certain molecules, e.g., once biology uses a set of compounds, it is predisposed to use similar ones and avoid dissimilar components. If this is true, we might expect biology elsewhere in the universe to be very similar to terrestrial biology, especially as there may be certain general entry points for environmentally common C, N or S-containing raw materials (e.g., CO_2_, CH_4_, N_2_, NH_3_, H_2_S, SO_2_, etc.) into the network of chemically allowed biotransformations.

The canonical AAs, if they cluster according to definable properties such as electrotopological metrics, may then represent a built-in teleological endpoint for evolution, in that the evolution of pathways for their formation also shapes their synthesis during biology’s exploration of fitness landscapes.

We note that there are possible relationships of the present study with the concept of substitution matrices [48,49], and a systematic exploration of these relationships may be a promising starting point for future work.

We presented here, for the first time, and for a very large dataset of XAAs, methodologies and results to select candidates for CAA substitutions in proteins based on structural similarity. The provided data can significantly refine the selection process of non-canonical amino acids for various applications in protein engineering (see SI section Applications of Xeno Amino Acids).

## Figures and Tables

**Table 1:** Abbreviations, short descriptions, references or definition, and sizes of the sets of amino acids (AAs) used in this study.

Abbreviation	Description	Reference/Definition	Size
CAAs	Canonical AAs (standard code)	e.g., Cleaves (2010) [5]	20
EAAs	Experimentally evaluated AAs	Josephson et al. (2005) [19] Hartman et al. (2007) [20]	105
GAAs	(computationally) Generated AAs	Ilardo et al. (2015) [13]	1913
PAAs	Purchasable AAs	www.emolecules.com	9518
AAAs	All AAs	EAA ∪ GAA ∪ PAA	11,302
XAAs	Xeno AAs	AAA\CAA	11,282

**Table 2:** Statistical parameters of TCs for CAAs, CAA × XAA, XAAs, and AAAs.

Parameter	CAA Pairs	CAA × XAA	XAA Pairs	AAA Pairs
Number of TCs	190	225,640	63,636,121	63,861,951
Minimum	0.11111	0.06579	0.06173	0.06173
First quartile	0.25767	0.13793	0.12500	0.12500
Median	0.32129	0.17647	0.15385	0.15385
Third quartile	0.38844	0.22642	0.19118	0.19149
90% quantile	0.46321	0.28125	0.23214	0.23214
99% quantile	0.61389	0.40000	0.33333	0.33333
Maximum	0.65000	0.75000	1.00000	1.00000
Mean	0.32803	0.18964	0.16369	0.16379
Std. deviation	0.11054	0.06860	0.05301	0.05309

**Table 3:** Tanimoto coefficients for CAA. Table cells are colored by a red–yellow–green scale, with red indicating low TC, and green high TC.

	G	A	S	P	V	T	C	I	L	N	D	Q	K	E	M	H	F	R	Y	W
G	1.000	0.333	0.350	0.208	0.286	0.273	0.333	0.280	0.292	0.318	0.381	0.259	0.286	0.320	0.241	0.242	0.206	0.194	0.200	0.152
A	0.333	1.000	0.450	0.192	0.611	0.579	0.429	0.478	0.500	0.409	0.409	0.333	0.310	0.346	0.357	0.265	0.265	0.250	0.257	0.196
S	0.350	0.450	1.000	0.172	0.391	0.375	0.571	0.370	0.500	0.545	0.545	0.444	0.414	0.462	0.414	0.353	0.353	0.333	0.343	0.261
P	0.208	0.192	0.172	1.000	0.172	0.167	0.167	0.147	0.152	0.161	0.161	0.139	0.132	0.143	0.132	0.143	0.116	0.111	0.114	0.111
V	0.286	0.611	0.391	0.172	1.000	0.650	0.375	0.542	0.440	0.360	0.360	0.300	0.281	0.310	0.323	0.243	0.243	0.231	0.237	0.184
T	0.273	0.579	0.375	0.167	0.650	1.000	0.360	0.520	0.423	0.346	0.346	0.290	0.273	0.300	0.313	0.237	0.237	0.225	0.231	0.180
C	0.333	0.429	0.571	0.167	0.375	0.360	1.000	0.357	0.480	0.522	0.522	0.429	0.400	0.444	0.400	0.382	0.343	0.324	0.333	0.255
I	0.280	0.478	0.370	0.147	0.542	0.520	0.357	1.000	0.414	0.345	0.345	0.294	0.278	0.303	0.314	0.275	0.275	0.233	0.268	0.212
L	0.292	0.500	0.500	0.152	0.440	0.423	0.480	0.414	1.000	0.462	0.462	0.387	0.364	0.400	0.452	0.316	0.316	0.300	0.308	0.240
N	0.318	0.409	0.545	0.161	0.360	0.346	0.522	0.345	0.462	1.000	0.636	0.519	0.387	0.429	0.387	0.333	0.333	0.351	0.324	0.250
D	0.381	0.409	0.545	0.161	0.360	0.346	0.522	0.345	0.462	0.636	1.000	0.414	0.387	0.481	0.387	0.371	0.371	0.316	0.361	0.277
Q	0.259	0.333	0.444	0.139	0.300	0.290	0.429	0.294	0.387	0.519	0.414	1.000	0.412	0.607	0.412	0.293	0.293	0.375	0.286	0.226
K	0.286	0.310	0.414	0.132	0.281	0.273	0.400	0.278	0.364	0.387	0.387	0.412	1.000	0.424	0.389	0.279	0.310	0.425	0.302	0.241
E	0.320	0.346	0.462	0.143	0.310	0.300	0.444	0.303	0.400	0.429	0.481	0.607	0.424	1.000	0.424	0.300	0.300	0.350	0.293	0.231
M	0.241	0.357	0.414	0.132	0.323	0.313	0.400	0.314	0.452	0.387	0.387	0.412	0.389	0.424	1.000	0.279	0.279	0.326	0.273	0.218
H	0.242	0.265	0.353	0.143	0.243	0.237	0.382	0.275	0.316	0.333	0.371	0.293	0.279	0.300	0.279	1.000	0.395	0.265	0.386	0.333
F	0.206	0.265	0.353	0.116	0.243	0.237	0.343	0.275	0.316	0.333	0.371	0.293	0.310	0.300	0.279	0.395	1.000	0.292	0.564	0.385
R	0.194	0.250	0.333	0.111	0.231	0.225	0.324	0.233	0.300	0.351	0.316	0.375	0.425	0.350	0.326	0.265	0.292	1.000	0.286	0.213
Y	0.200	0.257	0.343	0.114	0.237	0.231	0.333	0.268	0.308	0.324	0.361	0.286	0.302	0.293	0.273	0.386	0.564	0.286	1.000	0.327
W	0.152	0.196	0.261	0.111	0.184	0.180	0.255	0.212	0.240	0.250	0.277	0.226	0.241	0.231	0.218	0.333	0.385	0.213	0.327	1.000

**Table 4:** Tanimoto coefficients and set memberships of the most similar amino acids for each CAA.

CAA	TC	ID	EAA	GAA	PAA	CAA	TC	ID	EAA	GAA	PAA
**G**	0.450	G1		x		**N**	0.636	D	x	x	x
**A**	0.611	A1		x	x		0.583	N2		x	x
	0.611	V		x	x	**D**	0.636	N	x	x	x
**S**	0.600	S1		x	x		0.609	D2	x		
**P**	0.750	P1	x		x	**Q**	0.607	E	x	x	x
	0.680	P2			x		0.581	Q2			x
	0.654	P3			x	**K**	0.714	K1		x	x
**V**	0.700	V1		x		**E**	0.607	Q	x	x	x
	0.684	A1		x	x		0.586	E2			x
	0.667	V3.1			x	**M**	0.606	M1			x
	0.667	V3.2			x	**H**	0.590	H1			x
**T**	0.714	T1		x		**F**	0.610	F1		x	
	0.682	T2.1			x	**R**	0.737	R1			x
	0.682	T2.2		x			0.676	R2			x
**C**	0.619	C1		x	x		0.667	R3			x
**I**	0.667	I1			x		0.658	R4			x
**L**	0.643	L1			x	**Y**	0.649	Y1			x
						**W**	0.623	W1	x		

## Data Availability

All data for reproducing results of our amino acid similarity study are available in the SI file AASS.csv and described in the SI section *Detailed Results*. All software used for this study is publicly available for free, and is cited in Section 3 of the main text (KNIME, OpenBabel, R) and in the SI (DataWarrior).

## Supplementary Materials

The following supporting information can be downloaded at: https://www.mdpi.com/article/10.3390/ijms252212343/s1. All reference [50,51,52,53,54,55,56,57,58,59] in supplementary are cited in text.
