# Supplementary material for: Similarity Analysis of Computer-Generated and Commercial Libraries for Targeted Biocompatible Coded Amino Acid Replacement
Source: Int J Mol Sci. 2024 Nov 17;25(22):12343. doi: 10.3390/ijms252212343 (PMC11595000; doi:10.3390/ijms252212343)
Supplement: Supplementary file 1 [file ijms-25-12343-s001.zip › ijms-3305039-supplementary.pdf]

Supplementary Information

Similarity Analysis of Computer-Generated and Commercial Libraries for Targeted Biocompatible Coded Amino Acid Replacement

Markus Meringer[1], Gerardo M. Casanola-Martin[2], Bakhtiyor Rasulev[2,3], H. James Cleaves II[4,5,6,*]

1. German Aerospace Center (DLR), Department of Atmospheric Processors, Münchner Straße 20, 82234 Oberpfaffenhofen, 82234 Wessling, Germany, +49 8153 281412, markus.meringer@dlr.de
2. Department of Coatings and Polymeric Materials, North Dakota State University, Fargo, ND 58108, USA; gerardo.casanolamart@ndsu.edu (G.M.C.-M.); bakhtiyor.rasulev@ndsu.edu (B.R.)
3. Department of Chemistry, National University of Uzbekistan, Tashkent 100174, Uzbekistan
4. Department of Chemistry, Howard University, Washington DC 20059, USA
5. Earth-Life Science Institute, Tokyo Institute of Technology, 2-12-1-IE-1 Ookayama, Meguro-ku, Tokyo 152-8550, Japan
6. Blue Marble Space Institute for Science, 1001 4th Ave, Suite 3201, Seattle, WA 98154, USA
* Corresponding author: H. James Cleaves II, email: henderson.cleaves@howard.edu

Additional file includes

- CSV table (compressed) with detailed results including SMILES, set memberships, TCs and MDS coordinates: AASS.csv

Tanimoto Coefficients

For two bit-vectors a and b of equal length let $|a|$ denote the number of bits set in a, $a \wedge b$ the result of the bitwise "and" operation applied to a and b, and $a \vee b$ the result of the bitwise "or" operation. The Tanimoto coefficient of a and b is defined as

$$\mathrm{TC}(a, b) = |a \wedge b| \,/\, |a \vee b|.$$

Using the identity $|a \vee b| = |a| + |b| - |a \wedge b|$ the Tanimoto coefficient can also be written as

$$\mathrm{TC}(a, b) = |a \wedge b| \,/\, (|a| + |b| - |a \wedge b|) = (|a| + |b| - |a \vee b|) \,/\, |a \vee b|.$$

We note that $\mathrm{TC}(a, b) = 1$ if a and b have all bits in common (*i.e.* $a = b$) and $\mathrm{TC}(a, b) = 0$ if a and b have no bits in common (*i.e.* $|a \wedge b| = 0$).

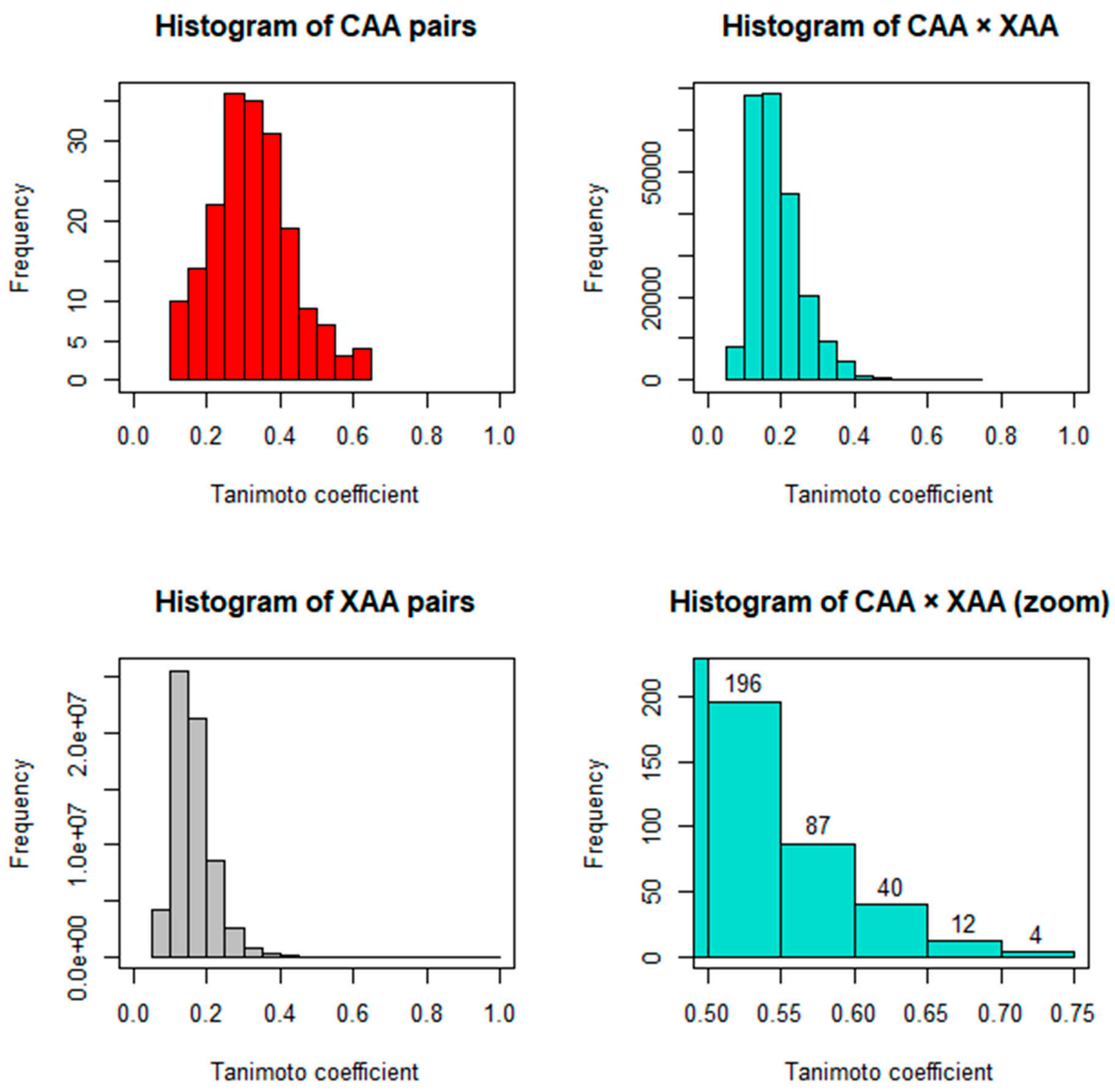

Figure S1. Histograms of TCs.

Detailed Results

AASS.csv offers all data for reproducing, further analyzing and visualizing the results of our amino acid similarity study. AASS.csv contains a table with 11,302 rows representing the amino acids and 27 columns providing the following information:

- NUM_ID with numerical identifiers of the amino acids corresponding to the row and column indices of the similarity matrix as sketched in Figure 3 of the main text
- SMILES with SMILES strings of the chemical structures
- CAA with one letter abbreviations for CAAs and empty entries for XAAs

- 3 columns (EAA, GAA and PAA) with entries 0/1 representing absence/presence in the corresponding sets
- EGP with strings of up to three letters E, G and P representing memberships in EAA, GAA and PAA. This is redundant information as the set memberships are already reported in the previous three columns. However, for some applications, such as DataWarrior [50] it is more handy to have this information combined in one column.
- 20 columns (TC_G, …, TC_W) with TCs based on ECFP6 for the 20 CAAs, indicated by the terminal letter of the column name, and ordered consistently with columns of Table 3 of the main text
- 2 columns (MDS_1 and MDS_2) with MDS coordinates as used for Figure 6 of the main text

This file contains all data to reproduce the rankings and the 2D projections of the main text and to inspect structures beyond those presented in Table 4 and Figure 5, as well as structures corresponding to data points of Figures 6. As an easy to handle, freely available software tool for that purpose we used DataWarrior [50]. It is able to open CSV files, display the table with the structures encoded by the SMILES string, sort rows by selected columns, display 2D views based on the MDS coordinates, zoom into areas of interest, and display structures of selected data points.

Choice of Fingerprints

Looking for the optimal radius for ECFP, initial experiments showed that with radius 4 there are many TCs of value 1 among AA pairs, *i.e.* ECFP4 would not provide sufficient resolution for our study. Increasing the radius to 6 reduces the amount of equal fingerprints dramatically: with ECFP6 only one pair of AAs with TC=1 is remaining (Figure S2).

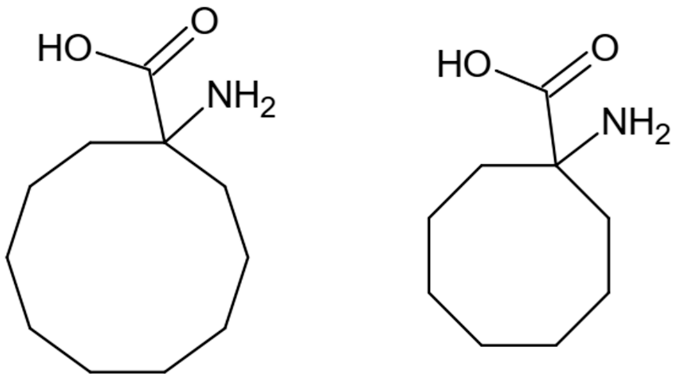

Figure S2: Pair of AAs with TC = 1.

Increasing the radius further to 8 would also result in different fingerprints for this pair, but we conclude that radius 6 offers sufficient resolution for our study.

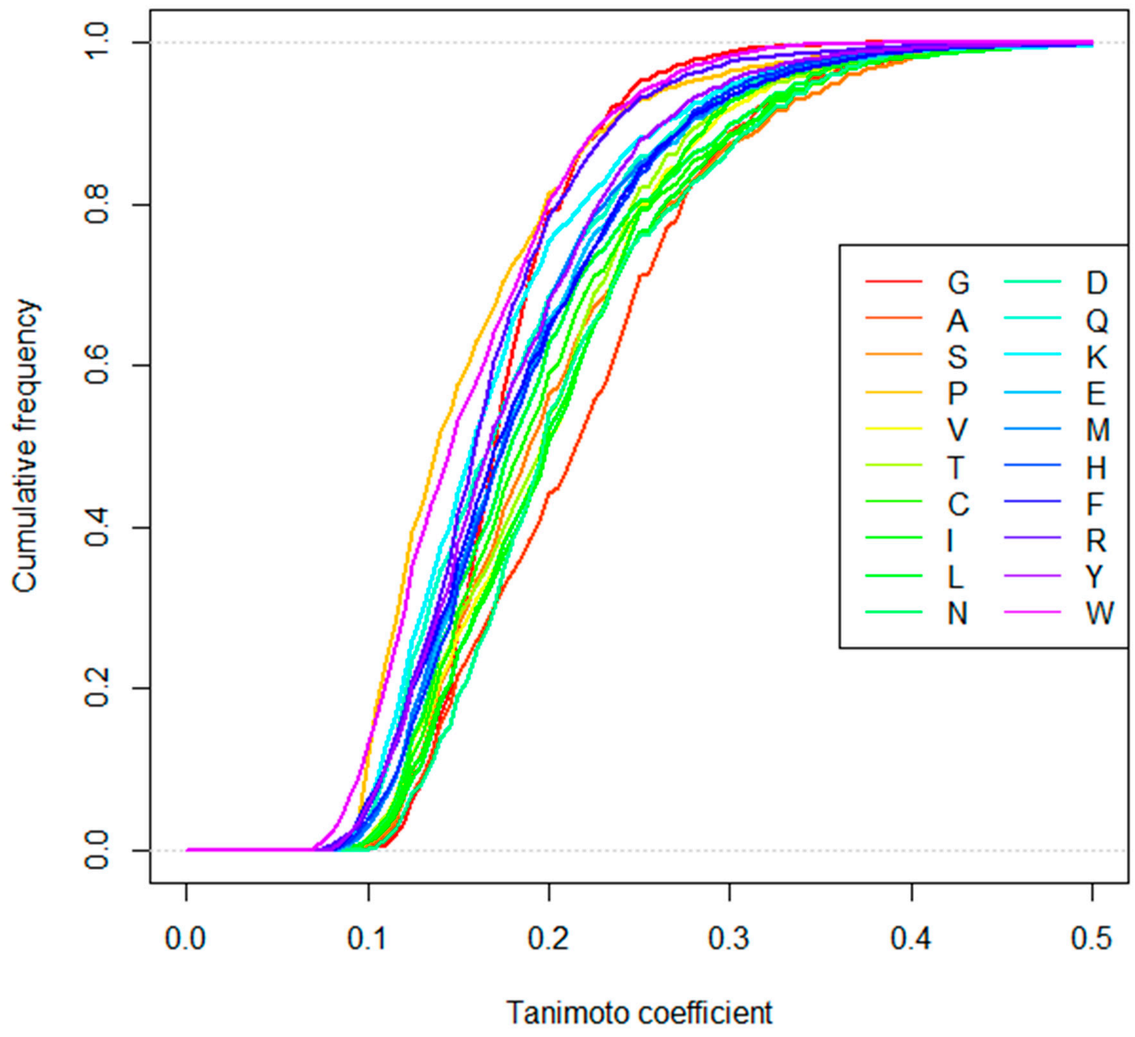

Figure S3. Cumulative relative frequencies of TCs for each CAA.

TC Outliers and Choice of TC Threshold

Figure S4 shows for each CAA a box plot of TCs with XAAs. Box plots, also known as box-and-whisker plots, offer useful criteria for detecting outliers in univariate data [51,52]. Boxes range from the first quartiles (Q1) to the third quartiles (Q3). The height of a box is called inter-quartile (IQ) range. Thick horizontal lines represent the medians. Whiskers mark the outer fences. The upper outer fence is defined by the uppermost data point below $Q3 + 3 \cdot IQ$, and analogously the lower outer fence as the lowermost data point above $Q1 - 3 \cdot IQ$. Data points outside the outer fences are called extreme outliers [53] and are depicted explicitly in box plots. There are no such outliers below the lower whiskers in Figure S4, but we see a total of 566 extreme outliers with high TC, and the corresponding structures would be good candidates for experimental evaluation.

In order to limit the number of structures to be presented in the main text to a moderate amount of about 30 compounds, we have chosen the following selection rules:

1. Report for each CAA the most similar XAA.
2. Show further highly ranked XAAs for each CAA with TC greater than 0.65.

The threshold of 0.65 is marked by a gray, dotted line in Figure S4, and coincides with the highest TC among CAAs, TC(V, T) = 0.65. Applying these two selection rules leads to a suitable number of 29 structures to be depicted in Figure 5, listed in Table 4 and discussed in the *Results* section of the main text.

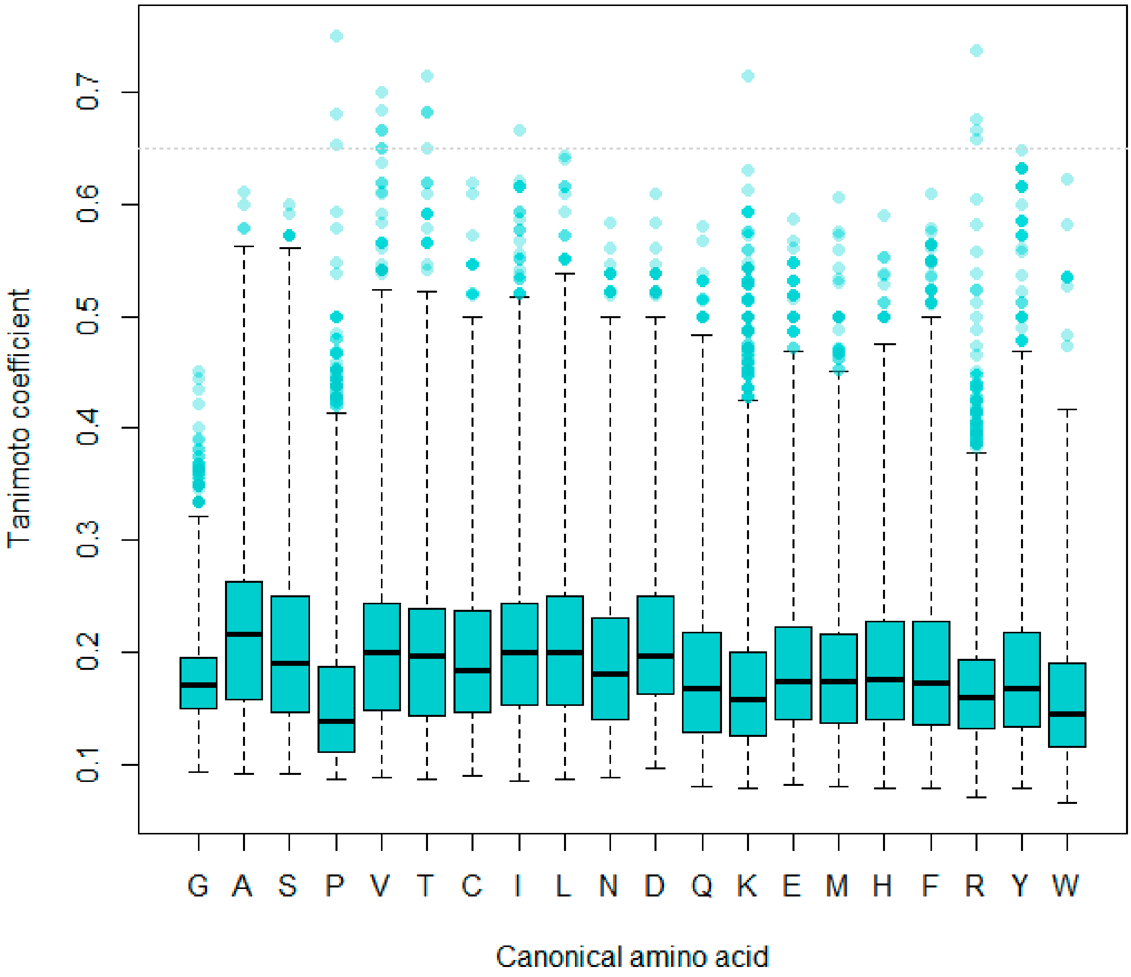

Figure S4. Box plots of TCs of CAAs with XAAs.

MDS Quality

In order to assess the quality of the similarity-based 2D projection of our AA chemical space, we visualized the correlation of 2D Euclidean Distances (ED) and Tanimoto Distances (TD) in Figures S5 and S6. Figure S5 shows this correlation as a scatterplot for pairs of CAAs (red dots) and CAA × XAA (turquoise dots). For CAAs the TDs and EDs are explicitly given in Tables S1 and S2. The good correlation of ED and TD is clearly visible in Figure S5 and numerically confirmed by correlation coefficients of 0.75678 for CAA × XAA and 0.91517 for CAA pairs. In order to visualize the correlation of ED and TD for the entire dataset, we show a 2D histogram in Figure S6 and computed the correlation coefficient of 0.72658. All correlation coefficients are summarized in Table S3.

Finally, we checked how much better the correlation of ED and TD would become if we applied classical MDA to CAA alone. The resulting 2D layout is depicted in Figure S7, a scatterplot with ED versus TD in Figure S8, and the correlation coefficient is 0.95995.

Table S4. Color-coded matrix of Tanimoto distances for CAA

	G	A	S	P	V	T	C	I	L	N	D	Q	K	E	M	H	F	R	Y	W
G	0.000	2.000	1.857	3.800	2.500	2.667	2.000	2.571	2.429	2.143	1.625	2.857	2.500	2.125	3.143	3.125	3.857	4.143	4.000	5.571
A	2.000	0.000	1.222	4.200	0.636	0.727	1.333	1.091	1.000	1.444	1.444	2.000	2.222	1.889	1.800	2.778	2.778	3.000	2.889	4.111
S	1.857	1.222	0.000	4.800	1.556	1.667	0.750	1.700	1.000	0.833	0.833	1.250	1.417	1.167	1.417	1.833	1.833	2.000	1.917	2.833
P	3.800	4.200	4.800	0.000	4.800	5.000	5.000	5.800	5.600	5.200	5.200	6.200	6.600	6.000	6.600	6.000	7.600	8.000	7.800	8.000
V	2.500	0.636	1.556	4.800	0.000	0.538	1.667	0.846	1.273	1.778	1.778	2.333	2.556	2.222	2.100	3.111	3.111	3.333	3.222	4.444
T	2.667	0.727	1.667	5.000	0.538	0.000	1.778	0.923	1.364	1.889	1.889	2.444	2.667	2.333	2.200	3.222	3.222	3.444	3.333	4.556
C	2.000	1.333	0.750	5.000	1.667	1.778	0.000	1.800	1.083	0.917	0.917	1.333	1.500	1.250	1.500	1.615	1.917	2.083	2.000	2.917
I	2.571	1.091	1.700	5.800	0.846	0.923	1.800	0.000	1.417	1.900	1.900	2.400	2.600	2.300	2.182	2.636	2.636	3.300	2.727	3.727
L	2.429	1.000	1.000	5.600	1.273	1.364	1.083	1.417	0.000	1.167	1.167	1.583	1.750	1.500	1.214	2.167	2.167	2.333	2.250	3.167
N	2.143	1.444	0.833	5.200	1.778	1.889	0.917	1.900	1.167	0.000	0.571	0.929	1.583	1.333	1.583	2.000	2.000	1.846	2.083	3.000
D	1.625	1.444	0.833	5.200	1.778	1.889	0.917	1.900	1.167	0.571	0.000	1.417	1.583	1.077	1.583	1.692	1.692	2.167	1.769	2.615
Q	2.857	2.000	1.250	6.200	2.333	2.444	1.333	2.400	1.583	0.929	1.417	0.000	1.429	0.647	1.429	2.417	2.417	1.667	2.500	3.417
K	2.500	2.222	1.417	6.600	2.556	2.667	1.500	2.600	1.750	1.583	1.583	1.429	0.000	1.357	1.571	2.583	2.231	1.353	2.308	3.154
E	2.125	1.889	1.167	6.000	2.222	2.333	1.250	2.300	1.500	1.333	1.077	0.647	1.357	0.000	1.357	2.333	2.333	1.857	2.417	3.333
M	3.143	1.800	1.417	6.600	2.100	2.200	1.500	2.182	1.214	1.583	1.583	1.429	1.571	1.357	0.000	2.583	2.583	2.071	2.667	3.583
H	3.125	2.778	1.833	6.000	3.111	3.222	1.615	2.636	2.167	2.000	1.692	2.417	2.583	2.333	2.583	0.000	1.529	2.769	1.588	2.000
F	3.857	2.778	1.833	7.600	3.111	3.222	1.917	2.636	2.167	2.000	1.692	2.417	2.231	2.333	2.583	1.529	0.000	2.429	0.773	1.600
R	4.143	3.000	2.000	8.000	3.333	3.444	2.083	3.300	2.333	1.846	2.167	1.667	1.353	1.857	2.071	2.769	2.429	0.000	2.500	3.692
Y	4.000	2.889	1.917	7.800	3.222	3.333	2.000	2.727	2.250	2.083	1.769	2.500	2.308	2.417	2.667	1.588	0.773	2.500	0.000	2.056
W	5.571	4.111	2.833	8.000	4.444	4.556	2.917	3.727	3.167	3.000	2.615	3.417	3.154	3.333	3.583	2.000	1.600	3.692	2.056	0.000

Table S1. Color-coded matrix of Euclidean distances among CAA 2D coordinates obtained by MDA

	G	A	S	P	V	T	C	I	L	N	D	Q	K	E	M	H	F	R	Y	W
G	0.000	2.224	2.310	4.758	2.544	2.678	2.368	1.930	2.064	2.544	2.086	3.180	3.224	2.490	2.540	3.452	3.980	2.229	3.867	5.247
A	2.224	0.000	0.333	5.228	0.321	0.458	0.333	0.671	0.591	0.426	1.286	1.113	1.072	0.969	0.711	2.074	2.820	1.326	2.642	3.565
S	2.310	0.333	0.000	5.551	0.395	0.470	0.064	0.495	0.366	0.241	1.027	0.893	0.915	0.639	0.383	1.744	2.487	1.044	2.310	3.274
P	4.758	5.228	5.551	0.000	5.392	5.489	5.560	5.607	5.643	5.629	6.168	6.233	6.088	6.146	5.935	7.289	8.014	6.281	7.845	8.729
V	2.544	0.321	0.395	5.392	0.000	0.139	0.344	0.874	0.756	0.275	1.420	0.861	0.780	0.972	0.654	1.979	2.735	1.429	2.552	3.353
T	2.678	0.458	0.470	5.489	0.139	0.000	0.409	0.963	0.835	0.279	1.472	0.748	0.648	0.978	0.649	1.925	2.682	1.467	2.497	3.246
C	2.368	0.333	0.064	5.560	0.344	0.409	0.000	0.557	0.426	0.179	1.077	0.845	0.857	0.659	0.379	1.743	2.491	1.087	2.312	3.248
I	1.930	0.671	0.495	5.607	0.874	0.963	0.557	0.000	0.144	0.724	0.640	1.265	1.346	0.607	0.613	1.731	2.419	0.711	2.259	3.424
L	2.064	0.591	0.366	5.643	0.756	0.835	0.426	0.144	0.000	0.587	0.695	1.124	1.202	0.521	0.476	1.665	2.373	0.741	2.207	3.325
N	2.544	0.426	0.241	5.629	0.275	0.279	0.179	0.724	0.587	0.000	1.195	0.693	0.684	0.704	0.380	1.711	2.466	1.188	2.283	3.142
D	2.086	1.286	1.027	6.168	1.420	1.472	1.077	0.640	0.695	1.195	0.000	1.478	1.640	0.616	0.891	1.366	1.948	0.143	1.812	3.164
Q	3.180	1.113	0.893	6.233	0.861	0.748	0.845	1.265	1.124	0.693	1.478	0.000	0.232	0.861	0.658	1.338	2.074	1.403	1.886	2.499
K	3.224	1.072	0.915	6.088	0.780	0.648	0.857	1.346	1.202	0.684	1.640	0.232	0.000	1.028	0.771	1.568	2.299	1.578	2.111	2.644
E	2.490	0.969	0.639	6.146	0.972	0.978	0.659	0.607	0.521	0.704	0.616	0.861	1.028	0.000	0.330	1.148	1.870	0.551	1.700	2.818
M	2.540	0.711	0.383	5.935	0.654	0.649	0.379	0.613	0.476	0.380	0.891	0.658	0.771	0.330	0.000	1.364	2.112	0.855	1.933	2.908
H	3.452	2.074	1.744	7.289	1.979	1.925	1.743	1.731	1.665	1.711	1.366	1.338	1.568	1.148	1.364	0.000	0.757	1.223	0.573	1.809
F	3.980	2.820	2.487	8.014	2.735	2.682	2.491	2.419	2.373	2.466	1.948	2.074	2.299	1.870	2.112	0.757	0.000	1.811	0.188	1.557
R	2.229	1.326	1.044	6.281	1.429	1.467	1.087	0.711	0.741	1.188	0.143	1.403	1.578	0.551	0.855	1.223	1.811	0.000	1.673	3.021
Y	3.867	2.642	2.310	7.845	2.552	2.497	2.312	2.259	2.207	2.283	1.812	1.886	2.111	1.700	1.933	0.573	0.188	1.673	0.000	1.557
W	5.247	3.565	3.274	8.729	3.353	3.246	3.248	3.424	3.325	3.142	3.164	2.499	2.644	2.818	2.908	1.809	1.557	3.021	1.557	0.000

Table S2. Correlation coefficients *r* of Euclidean and Tanimoto distances and numbers of entries *n* of the distance matrices considered

	**CAA pairs**	CAA × XAA	**XAA pairs**	**AAA pairs**
n	190	225,640	63,636,121	63,861,951
r	0.91517	0.75678	0.72674	0.72658

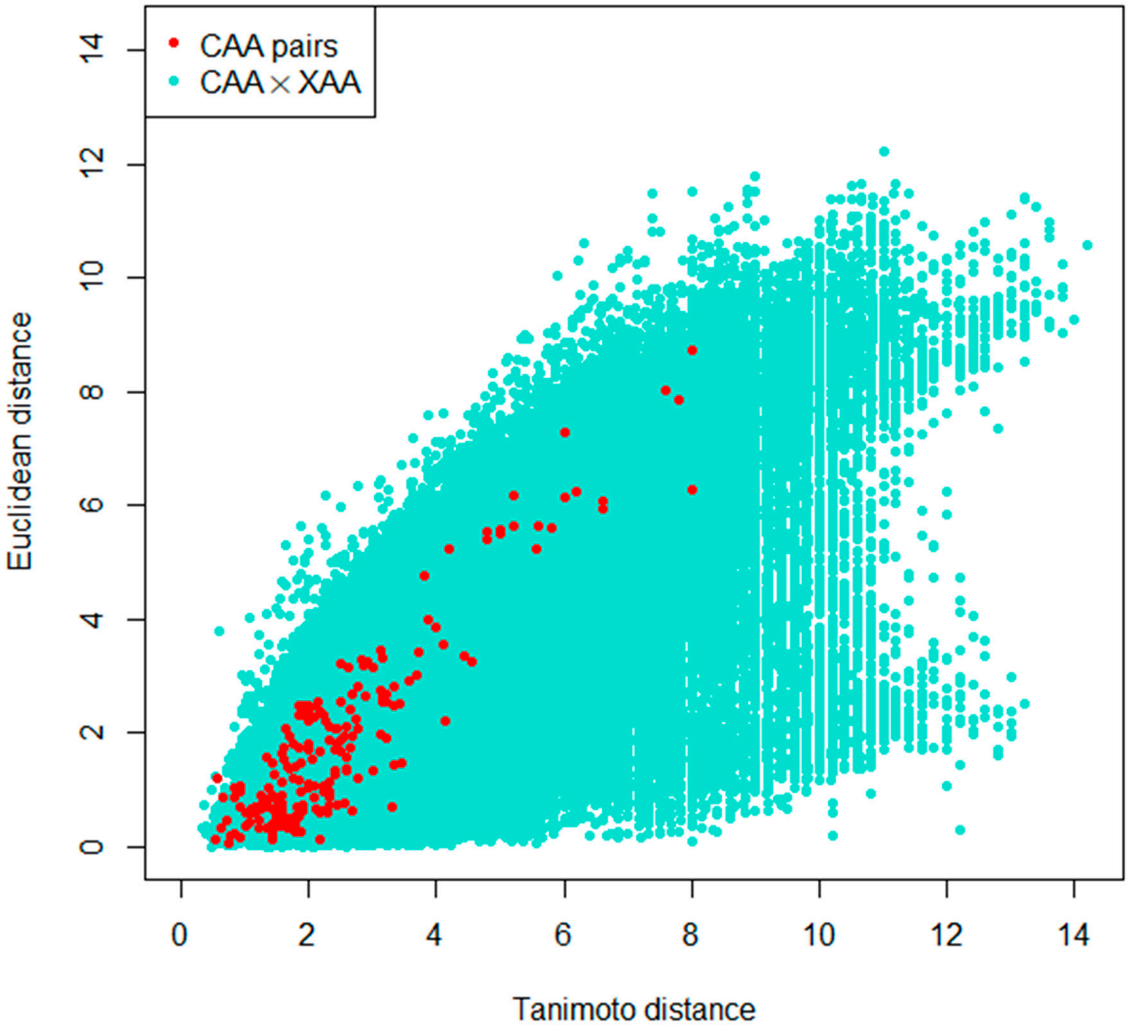

Figure S5. Scatterplot of Euclidean distances versus Tanimoto distances of CAA pairs and CAA × XAA.

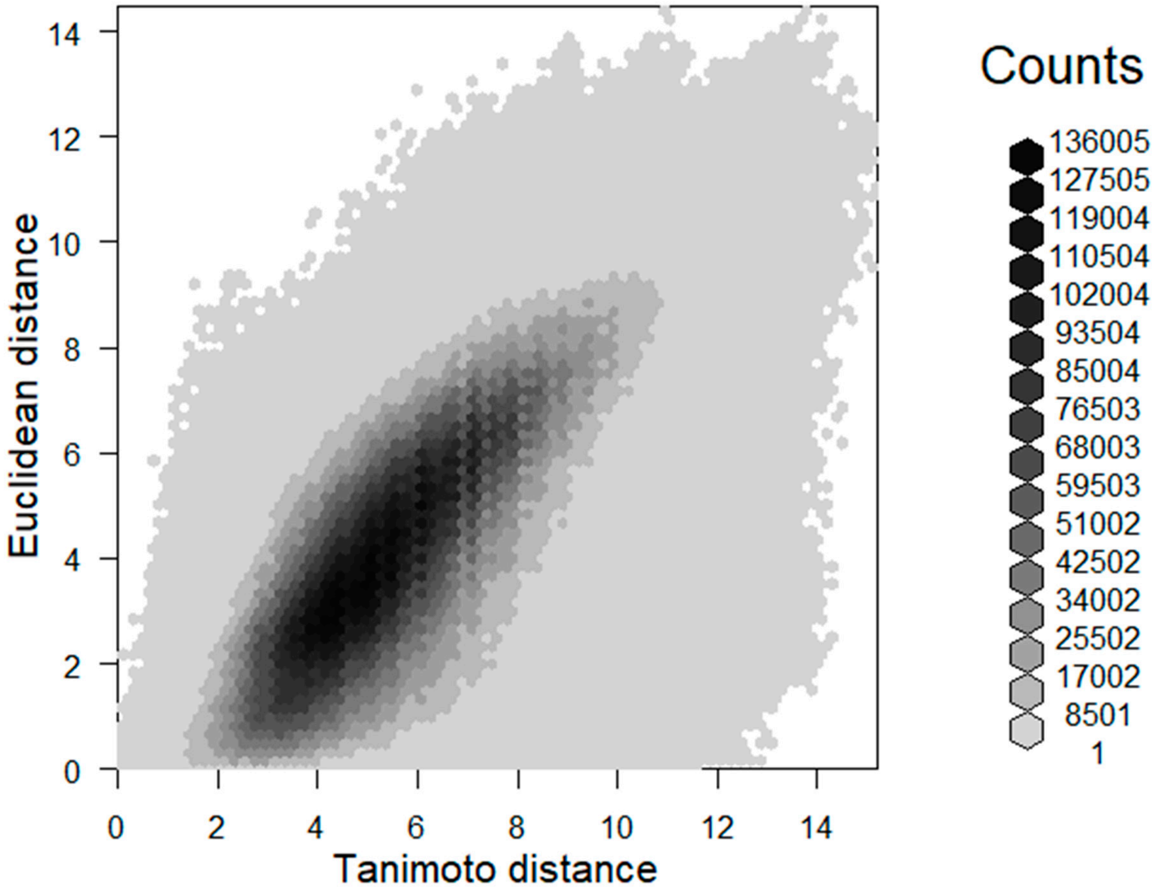

Figure S6. Euclidean distances versus Tanimoto distances of AAA pairs.

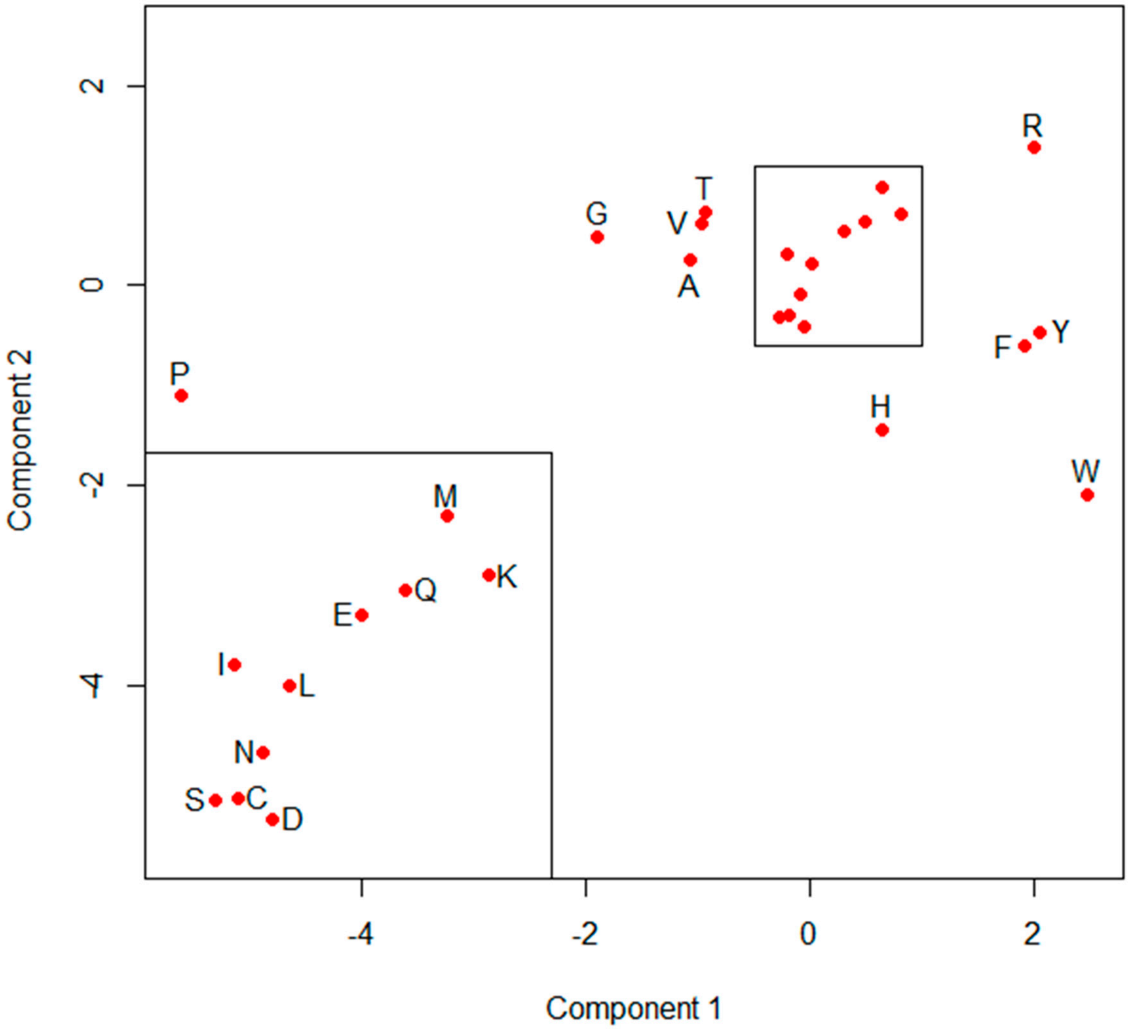

Figure S7. Classical MDS for CAA alone.

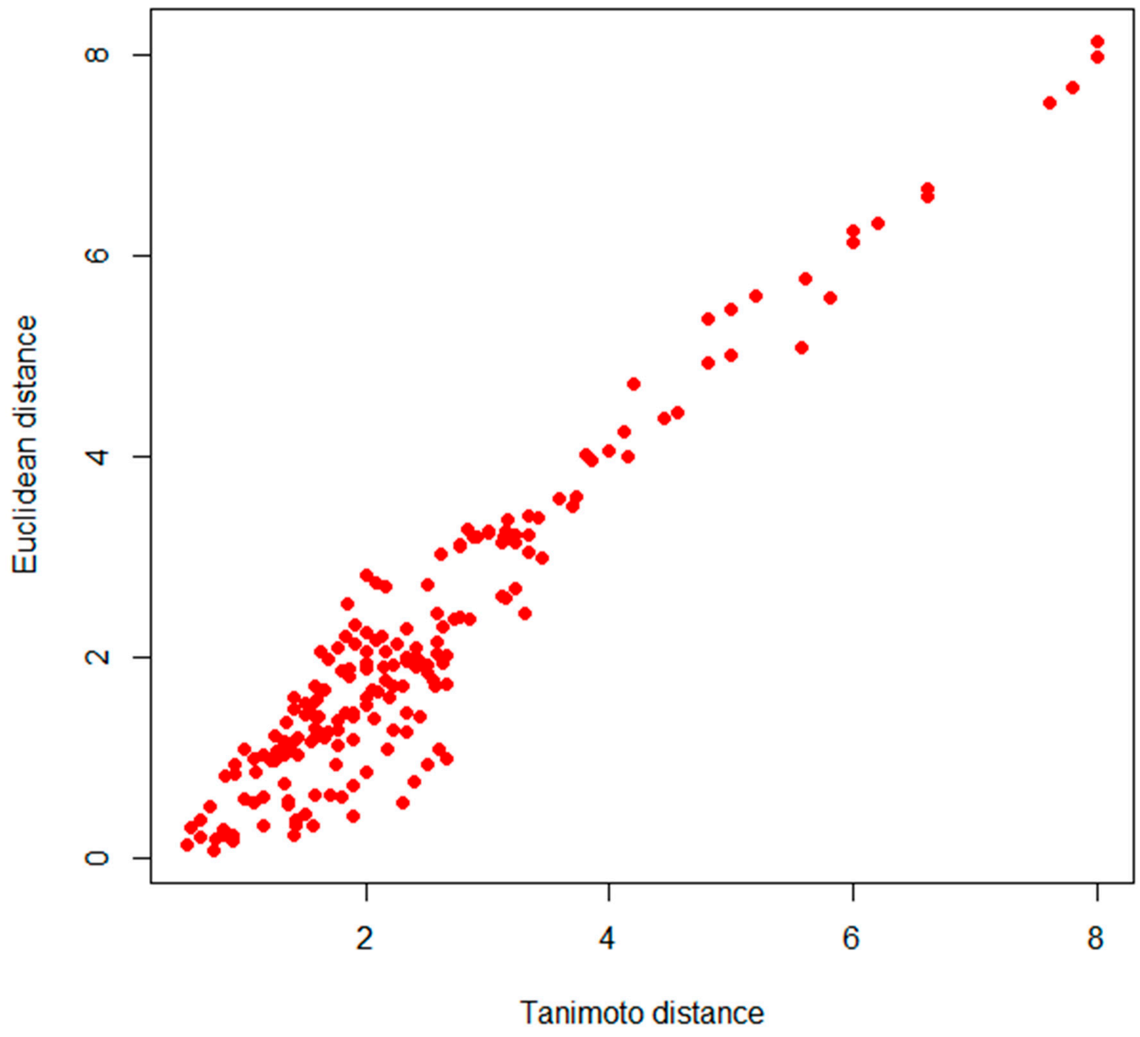

Figure S8. ED versus TD for classical MDS of CAA alone.

Table S3. Most important R functions used in this study, together with the including R package name and version, and short descriptions.

R function	R package	Description
parse.smiles	rcdk 3.8.1	parse SMILES strings into molecule objects
get.fingerprint	rcdk 3.8.1	compute molecular fingerprints
fp.sim.matrix	fingerprint 3.5.7	calculate a similarity matrix for a set of fingerprints
ecdf	stats 4.3.1	compute empirical cumulative distribution function
curve	graphics 4.3.1	draw function plots

cmdscale	stats 4.3.1	classical multidimensional scaling
hist	stats 4.3.1	compute a histogram
boxplot	graphics 4.3.1	produce box-and-whisker plots
hexbin	hexbin 1.28.3	bivariate binning into hexagon cells

Applications of Xeno Amino Acids

Applications of non-canonical AAs are described widely in the literature, see *e.g.* [54,55,56, 57,58,59].

References

50. Sander, T. Freyss, J., von Korff, M. & Rufener, C. DataWarrior: An open-source program for chemistry aware data visualization and analysis. *Journal of Chemical Information and Modeling* **2015**, *55*: 460-473 (download available at https://openmolecules.org/datawarrior, accessed July 20, 2024).

51. Tukey, J. W. Exploratory data analysis **1997**, (Vol. 2, pp. 131-160). Reading, MA: Addison-Wesley.

52. DuToit, S. H., Steyn, A. G. W., & Stumpf, R. H. Graphical exploratory data analysis. Springer Science & Business Media **2012**.

53. Heckert, N., Filliben, J., Croarkin, C., Hembree, B., Guthrie, W., Tobias, P. and Prinz, J. (2002), Handbook 151: NIST/SEMATECH e-Handbook of Statistical Methods, NIST Interagency/Internal Report (NISTIR), National Institute of Standards and Technology, Gaithersburg, MD (https://www.itl.nist.gov/div898/handbook/prc/section1/prc16.htm, accessed July 20, 2024).

54. Hohsaka, T. and Sisido, M. Incorporation of non-natural amino acids into proteins. *Current Opinion in Chemical Biology* **2002**, *6*(6), 809-815.

55. Link, A. J., Mock, M. L., & Tirrell, D. A. Non-canonical amino acids in protein engineering. *Current Opinion in Biotechnology* **2003**, 14(6), 603-609.

56. Wang, L. and Schultz, P. G. Expanding the genetic code. *Angewandte Chemie International Edition* **2005**, *44*(1), 34-66.

57. Liu, C. C. and Schultz, P. G. Adding new chemistries to the genetic code. *Annual Review of Biochemistry* **2010**, 79(1), 413-444.

58. Agostini, F., Völler, J. S., Koksch, B., Acevedo-Rocha, C. G., Kubyshkin, V., and Budisa, N. Biocatalysis with unnatural amino acids: enzymology meets xenobiology. *Angewandte Chemie International Edition* **2017**, *56*(33), 9680-9703.

59. Bell, E. L., Finnigan, W., France, S. P., Green, A. P., Hayes, M. A., Hepworth, L. J., ... and Flitsch, S. L. Biocatalysis. *Nature Reviews Methods Primers* **2021**, *1*(1), 1-21.
